# Genome-Wide Identification and Expression Analysis of the bHLH Transcription Factor Family in Wintersweet (*Chimonanthus praecox*)

**DOI:** 10.3390/ijms241713462

**Published:** 2023-08-30

**Authors:** Hafiz Muhammad Kamran, Xuemei Fu, Huabo Wang, Nan Yang, Longqing Chen

**Affiliations:** Yunnan Province Engineering Research Center for Functional Flower Resources and Industrialization, College of Landscape Architecture and Horticulture Sciences, Southwest Forestry University, Kunming 650224, China; silversand15@gmail.com (H.M.K.);

**Keywords:** bHLH family, Wintersweet, expression analysis, protein–protein interactions

## Abstract

Wintersweet (*Chimonanthus praecox* (L.) Link, Calycanthaceae) is an esteemed ornamental flowering shrub known for its distinct blooming period in winter, vibrant color petals, and captivating floral fragrance. Basic helix-loop-helix (bHLH) transcription factors (TFs) play pivotal roles as key regulators in secondary metabolites biosynthesis, growth, and development in plants. However, the systematic analysis of the bHLH family members and their role in the regulation of floral traits in Wintersweet remains insufficiently understood. To bridge this knowledge gap, we conducted a comprehensive genome-wide analysis of the *C. praecox* bHLH (*CpbHLH*) gene family, identifying a total of 131 *CpbHLH* genes across 11 chromosomes. Phylogenetic analysis classified these *CpbHLH* genes into 23 subfamilies, wherein most members within the same subfamily exhibited analogous intron/exon patterns and motif composition. Moreover, the expansion of the *CpbHLH* gene family was primarily driven by segmental duplication, with duplicated gene pairs experiencing purifying selection during evolution. Transcriptomic analysis revealed diverse expression patterns of *CpbHLH* genes in various tissues and distinct stages of Wintersweet flower development, thereby suggesting their involvement in a diverse array of physiological processes. Furthermore, yeast 2-hybrid assay demonstrated interaction between CpbHLH25 and CpbHLH59 (regulators of floral scent and color) as well as with CpbHLH112 and CpMYB2, suggesting potential coordinately regulation of secondary metabolites biosynthesis in Wintersweet flowers. Collectively, our comprehensive analysis provides valuable insights into the structural attributes, evolutionary dynamics, and expression profiles of the *CpbHLH* gene family, laying a solid foundation for further explorations of the multifaceted physiological and molecular roles of bHLH TFs in Wintersweet.

## 1. Introduction

Transcription factors (TFs) are pivotal regulators that orchestrate a wide range of biological processes using the activation or repression of target gene expression [1,2,3,4,5]. TFs belong to different families, such as MYBs (myeloblastosis), bHLHs (basic helix-loop-helix transcription factors), bZIPs (basic leucine zippers), and WRKY [6,7,8,9]. Among these families, bHLHs constitute one of the largest TF families in plants [10]. The bHLH family members typically contain a conserved bHLH domain that consists of approximately 60 amino acids, which is divided into two functionally distinct regions: the basic region and the helix-loop-helix (HLH) region [11]. The basic region, which spans approximately 15 amino acids and is situated at the N-terminus of the bHLH domain, encourages binding to the cis-elements in DNA. On the other hand, the HLH region, composed of around 40 amino acids and located at the C-terminus of the bHLH domain, facilitates the formation of protein complexes, including homodimers and heterodimers [12,13]. Additionally, in *Arabidopsis thaliana* (L.) Heynh. (Brassicaceae), certain atypical bHLHs have been identified and characterized, featuring a less conserved basic region that is essential for DNA binding [14,15].

The bHLH TFs are important regulators of various biological processes in plants, including secondary metabolism [16,17,18], plant growth and development [19,20], and stress tolerance [21,22,23]. The initial discovery of a plant bHLH TF was the R gene, which regulates the structural genes involved in anthocyanin formation [24]. bHLH TFs engage in interactions with MYB and WD40 repeat (WDR) TFs, forming a ternary complex known as MBW. This MBW complex plays a regulatory role in the transcription of genes involved in the biosynthesis of flavonoids in plants [25,26]. In blueberry fruit (*Vaccinium* spp.), *VcbHLHL1* stimulates anthocyanin accumulation and pigment development by interacting with *VcMYBL1* and *VcWDL2* [27]. Several bHLH genes related to anthocyanin biosynthesis have been functionally characterized in various plant species, such as *AtTT8*, *AtGL3,* and *AtEGL3* in *A. thaliana* [28] and *DcTT8* in *Dendrobium candidum* [29]. In *A. thaliana*, the HEC gene encoding bHLH TF was found to be involved in female reproductive tract development, depending on the level of loss in HEC function, infertility, and developmental abnormalities observed in plants [30]. BIGPETALp, another gene encoding bHLH TF, regulates petal growth by interfering in postmitotic cell expansion [31]. Recent research has identified *AabHLH112* as a positive regulator of sesquiterpenes biosynthesis (*β*-caryophyllene, *epi*-cedrol, and *β*-farnesene) in *Artemisia annua* L. (Asteraceae) [32]. In *Solanum lycopersicum* L. (Solanaceae), knockdown of *SlMYC1* resulted in the reduction in monoterpene content in leaf and stem trichome and sesquiterpenes (*β*-caryophyllene and *α*-humulene) in leaf trichome, while the content of *β*-caryophyllene and *α*-humulene increased in stem trichome, indicating differential regulation by *SlMYC1* [33]. Given the complex regulation of bHLH TFs in controlling various floral traits, it is crucial to continue studying this TF family and identify additional bHLH TFs and their mechanisms of regulation.

*Chimonanthus praecox*, commonly known as Wintersweet, has a long history of cultivation in China, dating back over a thousand years [34,35]. It is highly valued for its winter blooming period, bright yellow color, and intense fragrance, making it a popular ornamental plant [34]. Notably, the unique blooming season of Wintersweet suggests a potential for distinct molecular mechanisms governing flower development compared to spring-blooming plants [36,37]. Additionally, variation in the floral volatile profiles and pigment compositions among different Wintersweet genotypes further positions it as an ideal target for exploring floral traits in ornamental plants [38,39]. The genome-wide identification of bHLH transcription factors (TFs) holds great potential for comprehending their biological functions. However, the knowledge on the bHLH TF family in Wintersweet is limited, with only a few studies focusing on *CpbHLH1* [18], *CpTT8* [40], *CpbHLH13,* and *CpMYC2* [41]. To shed light on the functions of bHLH TFs in Wintersweet, a comprehensive genome-wide study was conducted using the recently published genome database [42]. This study analyzed various aspects of the *CpbHLH* TFs, including evolutionary history, chromosomal distribution, gene duplication, protein motifs, and gene structures. Furthermore, this study conducted an analysis of the expression patterns of *CpbHLH* genes using transcriptome data obtained from different organs of Wintersweet plants, as well as during various stages of flower development in two Wintersweet genotypes. The findings from this study provide a valuable resource for future investigations exploring the intricate relationship between *CpbHLH* genes and diverse floral characteristics within Wintersweet.

## 2. Results

### 2.1. Identification, Sequence Analysis, and Chromosomal Location of CpbHLHs

**Figure 1:**
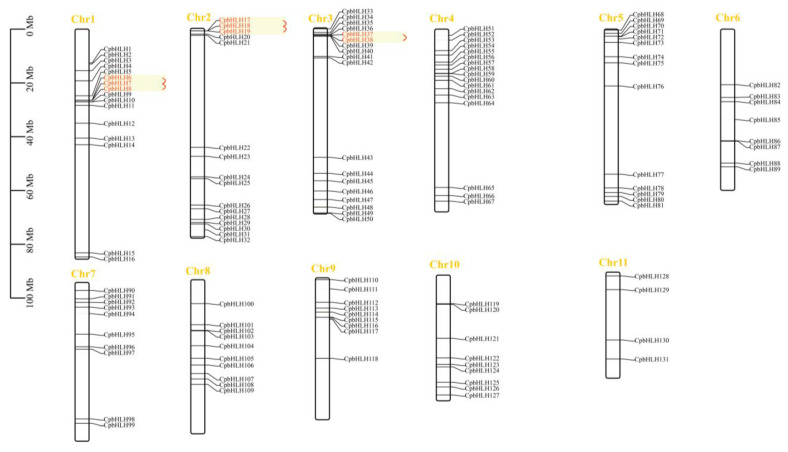
Schematic depiction of the chromosomal position of *CpbHLH* genes. At the top of each chromosome is the corresponding chromosome number. Tandem duplicates are indicated with red letters.

**Figure 2:**
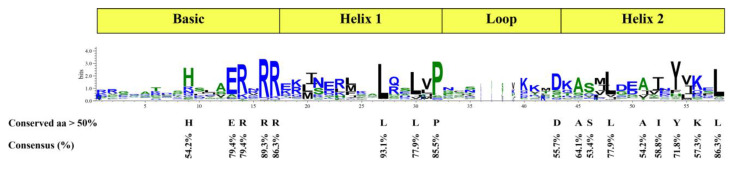
Depiction of conserved amino acid residues in the bHLH domain of the CpbHLH proteins. The height of each amino acid residue represents its degree of conservation.

In the Wintersweet genome, 131 putative *CpbHLH* genes were identified by combining the results of conserved domains and HMM identification. These *CpbHLH* genes were renamed *CpbHLH1* to *CpbHLH131* based on their distribution across different chromosomes. Next, we analyzed the physicochemical characteristics of the putative *CpbHLH*s and found diversities in open reading frames (ORFs), protein length, molecular weight (Mw), isoelectric point (PI), subcellular localization, and GRAVY. Specifically, the ORFs of the *CpbHLH* genes ranged from 273 to 2277 bp, and their corresponding protein products varied in length from 90 aa to 758 aa. The molecular weight (MW) and isoelectric point (PI) of *CpbHLH*s were found to range from 10218.55 to 82809.87 and 4.63 to 11.24, respectively (Table S1). Additionally, subcellular localization predictions revealed that all CpbHLH proteins, with the exception of CpbHLH36, CpbHLH38, CpbHLH99, CpbHLH76, CpbHLH63, CpbHLH73, CpbHLH120, CpbHLH31, CpbHLH54, CpbHLH19, CpbHLH17, CpbHLH18, CpbHLH121, CpbHLH103, CpbHLH80, CpbHLH122, CpbHLH82, CpbHLH9, CpbHLH11, CpbHLH126, CpbHLH16, CpbHLH88, CpbHLH107, and CpbHLH124, were localized within the nucleus (Table S1). The genome annotation information indicated that the 131 *CpbHLH* genes were located on eleven different chromosomes, with an uneven distribution of *CpbHLH* genes across each chromosome. Chromosome 3 contained the highest number of *CpbHLH* genes (18), followed by chromosome 4 with 17 *CpbHLH* genes, while chromosome 11 had the lowest number of *CpbHLH* genes (4) (Figure 1). The clustalW alignment of the 131 CpbHLH proteins revealed that the basic region and two helix regions of the bHLH domain were highly conserved in the CpbHLH proteins, except CpbHLH16, CpbHLH31, CpbHLH62, CpbHLH66, CpbHLH81, CpbHLH88, CpbHLH94, CpbHLH113, CpbHLH124, and CpbHLH126, which lacked one of these regions (Figure S1). In total, 17 amino acid residues of the bHLH domain were conserved with more than 50% consensus ratio, and 5 of them were conserved with a consensus ratio greater than 85%. Among the 17 conserved amino acid residues, five (His-9, Glu-13, Arg-14, Arg-16, and Arg-17) constitute the basic region, three (Leu-27, Leu-30, and Pro-32) were present in the helix 1 region, one (Asp-43) was in the loop region, and eight (Ala-45, Ser-46, Leu-48, Ala-51, Ile-52, Tyr-54, Lys-56, and Leu-58) were present in the helix 2 region (Figure 2).

### 2.2. Evolutionary Tree Analysis of CpbHLHs

**Figure 3:**
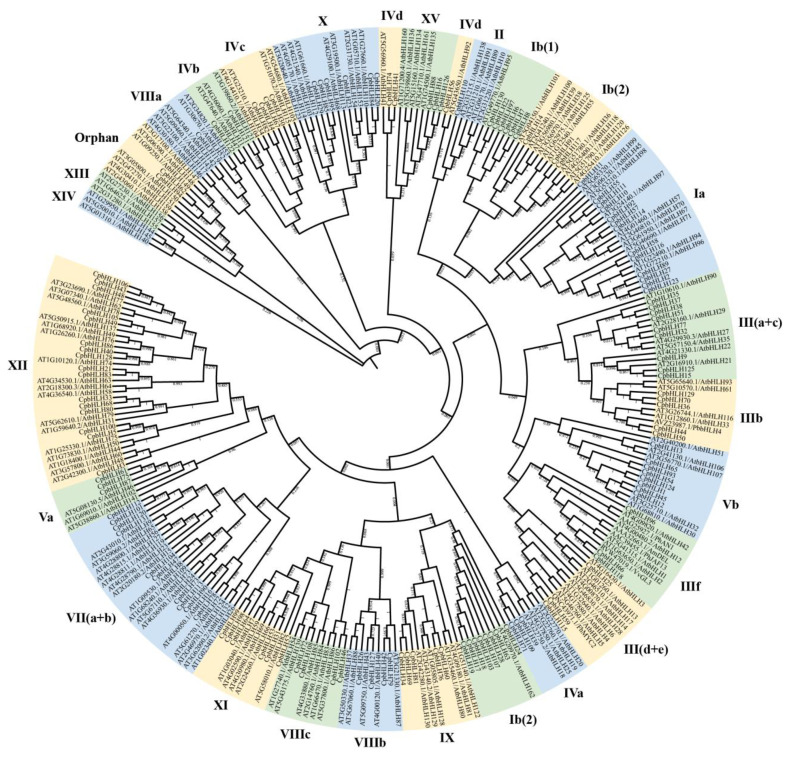
Phylogenetic tree analysis of *AtbHLH*s and *CpbHLH*s. The neighbor-joining (NJ) tree was generated using MEGA7 after aligning bHLH protein sequences with clustalW. The Roman numerals around the phylogenetic tree circle represent the subfamily name of bHLH genes.

To explore the evolutionary relationships and potential functions of *CpbHLH*s, a NJ phylogenetic tree was generated by aligning the full-length protein sequences of 131 *CpbHLH*s, 157 *AtbHLH*s and six functionally characterized bHLHs (*PhAN1*, *PhJAF13*, *VvGL3*, *PbbHLH4*, *AmDEL*, and *FhMYC2*). Based on the classification system of the *A*. *thaliana* bHLH family [43,44], all of these 294 bHLHs were grouped into 25 subfamilies in the phylogenetic tree (Figure 3). Among these subfamilies, 23 contained *CpbHLH*s, including Ia, Ib1, Ib2, II, III(a+c), IIIb, III(d+e), IIIf, IVa, IVb, IVc, IVd, Va, Vb, VII(a+b), VIIIa, VIIIb, VIIIc, IX, X, XI, XII, and XV. None of the *CpbHLH* genes clustered in the XIII and XIV subfamilies. The largest number of *CpbHLH* genes (16) was found in the XII subfamily, while the II and VIIIa subfamilies had the fewest number of *CpbHLH* genes, with only one *CpbHLH* gene in each (Figure 3). Moreover, the bHLH genes that could not cluster in any of the 25 subfamilies were termed orphans.

### 2.3. Gene Structure and Protein Motif Analysis of the CpbHLHs

**Figure 4:**
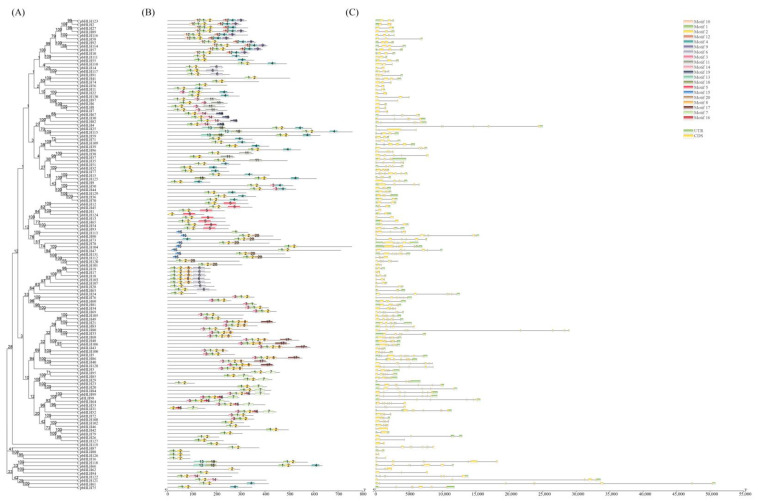
Phylogenetic tree, protein conserved motif, and intron-exon organization of Wintersweet bHLH family. (**A**) Phylogenetic tree relationship of *CpbHLH*s (**B**) Conserved motif analysis of CpbHLH proteins (**C**) exon intron organization analysis of bHLH genes. Yellow and green boxes indicate the exon and intron, respectively. Motif analysis was carried out by MEME suite, and the distinct 20 conserved motifs were represented with different color boxes.

The intron-exon organization and the protein motifs composition of *CpbHLH*s were analyzed to better understand their structure and functions. The MEME suite predicted 20 potential conserved motifs in 131 CpbHLH proteins, with motifs 1 and 2 being present in all CpbHLH proteins, except CpbHLH31, CpbHLH62, CpbHLH81, CpbHLH124, and CpbHLH113 which lacked one of these motifs, while the remaining conserved motifs were found only in certain gene sequences (Figure 4B). Motif 1 and 2 make up the bHLH domain, with motif 1 containing the basic region and helix 1 region, while, motif 2 comprising the loop and helix 2 region (Table S4). Most members of the same subfamily shared similar motif patterns. For instance, all members of subfamily IVc contained motifs 1, 2, 14, and 19, while Vb subfamily members contained motifs 1, 2, and 5. Moreover, most subfamily Ia members had motifs 10, 1, 2, 12, 4, and 9, while XI subfamily members contained motifs 3, 1, 2, 16, and 7 (Figure 4B). In addition, the number of exons ranged from 1 to 10 among 131 *CpbHLH* genes, with most genes having 2–8 exons, while *CpbHLH79* was intronless. *CpbHLH33* was found to have the highest number of exons (10) and introns (9) among all *CpbHLH* genes (Figure 4C). As expected, the majority of *CpbHLH* genes within the same subfamily had similar exon/intron organizations. For instance, most members of the IX, IVc, XI, and Vb subfamilies contained 6, 5, 7, and 2 exons, respectively (Figure 4C).

### 2.4. Pivotal cis-Acting Elements in the Promoter of CpbHLHs

TFs bind to cis-acting elements, which are usually located upstream of the 5′ end of a gene and are responsible for transcriptional regulation. Thus, to investigate the patterns of gene regulation, PlantCARE was utilized to analyze the 2 kb upstream sequence of the start codon of the *CpbHLH* genes, aiming to identify potential *cis*-acting elements. The promoters of all *CpbHLH*s exhibited a diverse range of *cis*-elements, which could be categorized into several groups, including development-related/organ-specific related (AACA_motif, CAT-box, O2-site, and so on), light-responsive elements (chs-CMA1a, GATA-motif, 3-AF1 binding site and so on), hormone-responsive elements (ABRE, AuxRR-core, GARE-motif and so on), stress-related elements (LTR, MBS, TC-rich repeats and so on), MYB-related elements, MYC elements, Binding site elements, and promoter-related elements. The presence of MRE, MBS, and MBS1 *cis*-acting elements in the promoter region of *CpbHLH* genes suggests that MYB TFs may play a role in regulating the transcription of bHLH genes in Wintersweet, potentially modulating the expression of downstream genes (Figure S2).

### 2.5. Gene Duplication Events and Synteny Analysis of CpbHLHs

**Figure 5:**
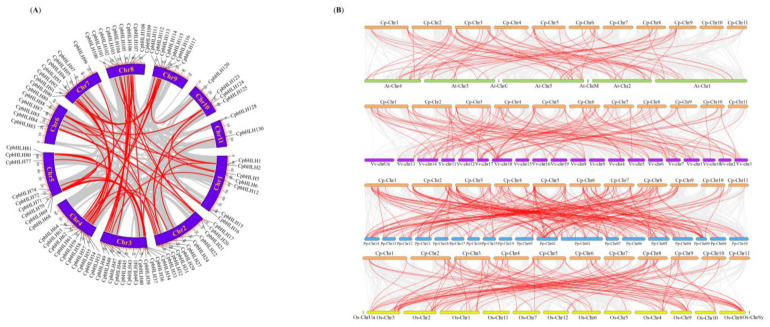
Syntenic analysis of *CpbHLH*s (**A**) Schematic representation of interchromosomal relationship of *CpbHLH* genes. Grey lines indicate all the syntenic relationships in the Wintersweet genome, while the red lines represent the segmental duplicated *CpbHLH* genes. (**B**) Synteny analysis of *Chimonanthus praecox* bHLHs with the *Arabidospsis thaliana*, *Vitis vinifera*, *Populus trichocarpa* (dicotyledon), and *Oryza sativa* (monocotyledon) bHLH genes. The grey lines in the background depict the collinear block between Wintersweet and other plant genomes, while the red line specifies the syntenic bHLH gene pairs.

To gain insight into the expansion mechanism of the *CpbHLH* gene family, we utilized Multiple Collinearity Scan (MCScanx) to examine gene duplication events in *C. praecox*. A total of 61 gene duplication events were identified, resulting in the formation of gene pairs within the *CpbHLH*s. Among these, five pairs (comprising 8 *CpbHLH* genes) were found to have arisen through tandem duplication, which occurred on chromosomes 1, 2, and 3 (Figure 1). Additionally, the remaining 56 pairs (consisting of 85 *CpbHLH* genes) appeared to be segmental duplicates distributed across all 11 chromosomes (Figure 5A). These findings indicate that gene duplication events likely contributed to the diversity of the *C. praecox* bHLH gene family, particularly segmental duplications. In addition, all the tandem duplicates and 55 segmental duplicats had a Ka/Ks value lower than 1, suggesting the influence of purifying selection during the evolutionary process (Table S2). The Ka/Ks value of 1 segmental duplicate (*CpbHLH37* and *CpbHLH109*) could not be calculated due to sequence divergence.

To explore the evolutionary relationship among bHLH genes of *A. thaliana*, *P. trichocarpa* Torr. and A.Gray ex. Hook. (Salicaceae), *V. vinifera* L. (Vitaceae) (dicotyledon), *O. sativa* L. (Poaceae) (monocotyledon), and *C. praecox*, collinearity analysis was carried out to identify orthologous bHLH genes across these species. Our analysis revealed a total of 68 orthologous gene pairs between *C. praecox* and *A. thaliana*, 187 pairs between *C. praecox* and *P. trichocarpa*, 109 pairs between *C. praecox* and *V. vinifera*, and 96 pairs between *C. praecox* and *O. sativa* (Figure 5B). Among the orthologous gene pairs involving *C. praecox* and *A. thaliana*, there were 48 *CpbHLH*s and 46 *AtbHLH*s, while the pairs between *C. praecox* and *P. trichocarpa* consisted of 81 *CpbHLH*s and 109 *PpbHLH*s. Furthermore, the pairs between *C. praecox* and *V. vinifera* comprised 74 *CpbHLH*s and 64 *VvbHLH*s, and the pairs between *C. praecox* and *O. sativa* included 62 *CpbHLH*s and 61 *OsbHLH* genes (Table S3). Additionally, several *CpbHLH* genes exhibited collinearity with more than three bHLH genes from other plant species, such as *CpbHLH101* (*C. praecox* and *O. sativa*), *CpbHLH34*, *CpbHLH99*, *CpbHLH69*, *CpbHLH100*, *CpbHLH64*, *CpbHLH95*, *CpbHLH26*, *CpbHLH20*, *CpbHLH21*, *CpbHLH81*, *CpbHLH5*, *CpbHLH93*, *CpbHLH85*, *CpbHLH84*, *CpbHLH83*, *CpbHLH30*, and *CpbHLH29* (*C. praecox* and *P. trichocarpa*) (Table S3).

### 2.6. Expression Analysis of CpbHLHs in Wintersweet

**Figure 6:**
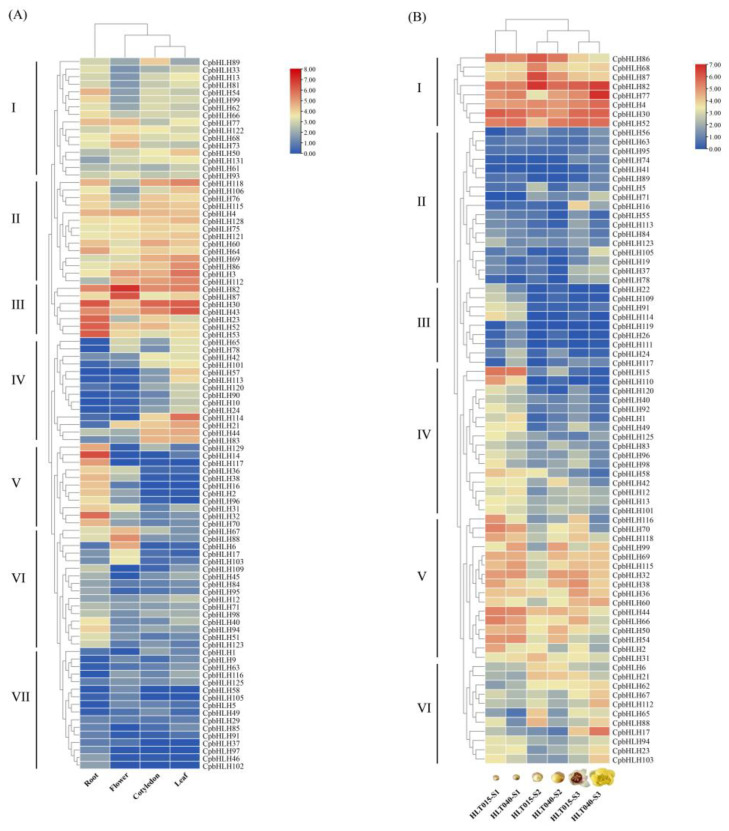
Expression analysis of *CpbHLH* genes. (**A**): Tissue specific expression of *CpbHLH*s in roots, leaves, flowers, and cotyledons. (**B**): Temporal expression profile of differentially expressed *CpbHLH*s in developing flowers of two Wintersweet genotypes flower. S1: green bud, S2: bud turning yellow, and S3: open flower.

To dissect the expression patterns of *CpbHLH* genes in different tissues of Wintersweet, the FPKM values of these genes were extracted from transcriptome data of four distinct organ samples, namely roots, leaves, cotyledon, and flowers. *CpbHLH* genes with FPKM values below 1 in all tissues were excluded, and the remaining genes were used to generate an expression heatmap. Out of the 131 *CpbHLH* genes, 94 showed expression in at least one of the four tissues analyzed in the transcriptome database. Cluster analysis was performed, resulting in the classification of the 94 *CpbHLH* genes into seven distinct groups based on their specific expression profiles (Figure 6A). Among 94 *CpbHLH* genes, few *CpbHLH*s exclusively expressed in one tissue, such as *CpbHLH117*, *CpbHLH14*, *CpbHLH16*, and *CpbHLH6*. Notably, *CpbHLH82*, *CpbHLH87*, *CpbHLH88*, *CpbHLH17*, *CpbHLH67*, *CpbHLH73*, *CpbHLH103* and *CpbHLH6* showed higher expression levels in the flower tissue, while *CpbHLH114*, *CpbHLH57* and *CpbHLH10* exhibited higher expression in leaves. Moreover, *CpbHLH32*, *CpbHLH14*, *CpbHLH16*, *CpbHLH70*, and *CpbHLH2* displayed specific expression patterns primarily in roots (Figure 6A).

To assess the temporal expression profile of *CpbHLH* genes in Wintersweet flower, six flower samples were subjected to DGE analysis, and the resulting data were analyzed using hierarchal cluster analysis, which grouped the 77 differentially expressed *CpbHLH* genes into six distinct clusters (Figure 6B). In Figure 6B, clusters 2, 3, and 4 demonstrated low expression levels across most of the tested samples, whereas cluster 1 exhibited high expression levels in the majority of the tested samples. Several *CpbHLH* genes exhibited significant temporal expression differences during flower development, such as *CpbHLH15*, *CpbHLH114*, *CpbHLH91*, *CpbHLH86*, *CpbHLH13*, *CpbHLH110* and *CpbHLH6* (Figure 6B) and their expression pattern was also similar in two genotypes. The expression of these genes peaked during the bud stages and decreased at the open flower stage during development. However, specific *CpbHLH* genes exhibited significant expression differences between the HLT015 and HLT040 genotypes during flower development. For instance, *CpbHLH68* and *CpbHLH87* were significantly upregulated at bud stage 2 in the HLT015 genotype compared to the HLT040 genotype. In contrast, *CpbHLH77* showed significant upregulation at bud stage 2 and the full open flower stage in the HLT040 genotype compared to the HLT015 genotype (Figure 6B). Furthermore, *CpbHLH17* and *CpbHLH112* exhibited high expression levels at the full open flower stage in the HLT040 genotype compared to the HLT015 genotype. Additionally, *CpbHLH118* and *CpbHLH66* were upregulated in the HLT015 genotype at the full open flower stage relative to the HLT040 genotype, displaying distinct expression patterns in both genotypes. These findings indicate that *CpbHLH* genes exhibit diverse expression patterns, suggesting potential functional divergence during flower development (Figure 6A,B).

**Figure 7:**
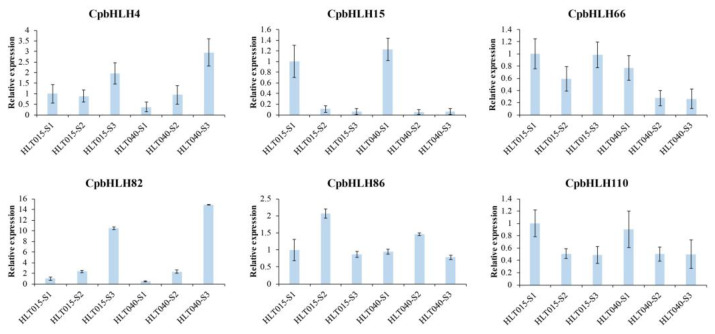
Validation of transcription level of *CpbHLH*s obtained from transcriptome database using RT-qPCR. *β*-actin was used as a reference gene to normalize the expression level of genes.

To validate the transcriptome data, the expression of six randomly selected *CpbHLH* genes was further analyzed using qRT-PCR. The qRT-PCR results revealed that the expression patterns of five genes, except *CpbHLH82,* during flower development were relatively consistent with the RNA-Seq. Results (Figure 7), thereby affirming the reliability of transcriptome data.

### 2.7. Protein–Protein Interaction

**Figure 8:**
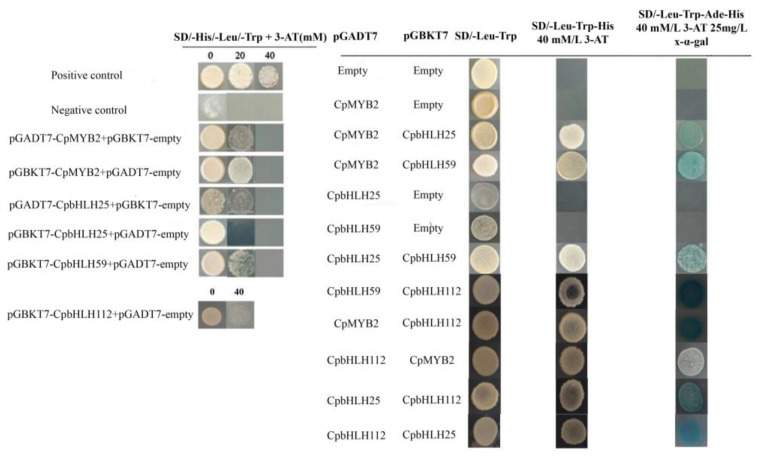
Evaluation of interactions between CpbHLH25, CpbHLH59, CpbHLH112, and CpMYB2 via Yeast two-hybrid system. SD/-Leu-Trp: Synthetic dropout media without leucine and tryptophan; SD/-Leu-Trp-His: Synthetic dropout media without leucine tryptophan and histidine; SD/-Leu-Trp-His-ade+3-AT+ X-α-Gal: Synthetic dropout media lacking leucine tryptophan, histidine and adenine but contain 3-amino-1,2,4-triazole and X-α-Gal.

Interactions among bHLH TFs and other TFs play a crucial role in the regulation of various biological processes. To investigate the potential interactions, yeast cells were transformed with different combinations of binding domain (BD) and activation domain (AD) fusion constructs. The yeast cells carrying BD-empty+AD-CpMYB2, BD-empty+AD-CpbHLH25, BD-empty+AD-CpbHLH59, and BD-empty+AD-empty did not exhibit any growth or interaction on SD/-Leu-Trp-His-Ade+3-AT+X-α-gal medium (Figure 8). In contrast, yeast cells carrying BD-CpbHLH25+AD-CpMYB2, BD-CpbHLH59+AD-CpMYB2, BD-CpbHLH59+AD-CpbHLH25, BD-CpbHLH112+AD-CpbHLH25, BD-CpbHLH112+AD-CpbHLH59, BD-CpbHLH112+AD-CpMYB2, BD-CpMYB2+AD-CpbHLH112, and BD-CpbHLH25+AD-CpbHLH112 displayed growth and turned blue (Figure 8), demonstrating the potential interactions between the tested TF pairs.

## 3. Discussion

The bHLH genes have demonstrated their involvement in a diverse range of physiological and biochemical processes (signaling, defense against stress, biosynthesis, and growth and development) in plants [18,20,45,46,47]. However, there has been limited research on the bHLH gene family in Wintersweet, with only a few specific genes (*CpbHLH1*, *CpTT8*, *CpMYC2*, and *CpbHLH13*) being functionally characterized [18,40,41]. The recent publication of the Wintersweet genome [42] has provided an opportunity to conduct a comprehensive analysis and characterization of the bHLH gene family at the genome level. Here, we identified 131 members belonging to the bHLH gene family in the Wintersweet genome. Interestingly, the number of bHLH genes in Wintersweet was found to be lower than those in *A. thaliana* (162) [11], *O. sativa* (183) [48], and *Malus domestica* (Suckow) Borkh. (Rosaceae) (175) [49] but higher than *Prunus avium* L. (Rosaceae) (66) [50] and *Carthamus tinctorius* L. (Asteraceae) (41) [51]. The variation in the number of bHLH genes among different species may be attributed to events such as genome/gene duplication or differences in genome size [52,53,54].

The classification of bHLH genes in plants varies across different species. For example, in *Prunus mume* (Siebold) Siebold and Zucc. (Rosaceae), 100 *PmbHLH* genes were categorized into 21 subfamilies, while in *Camellia sinensis* (L.) Kuntze (Theaceae), 134 *CsbHLH*s were divided into 17 subfamilies [55,56]. In our study, the *CpbHLH* genes clustered into 23 subfamilies and lacked members in XIII and XIV subfamilies (Figure 3), suggesting the possible loss of these genes during the evolution of Wintersweet. Further analysis of protein motifs and gene structures revealed similarities among most of the *CpbHLH* genes within the same subfamily, indicating their shared evolutionary origins and potentially similar physiological functions (Figure 4). The conservation of protein motifs 1 and 2, which constitute the DNA binding and protein dimerization region of the bHLH domain [57,58], was observed across *CpbHLH* genes. However, the composition of the remaining motifs was unique and conserved within subfamilies (Figure 4B). These variations in conserved motifs facilitate the categorization of proteins into subfamilies and reflect the specific functions carried out by each subfamily [59]. Moreover, insight into the evolution of gene families can be gained by analyzing gene structure [60]. *CpbHLH* genes exhibit variation in the number of introns ranging between 0 and 10, indicating the possibility of intron gain and loss that may contribute to the variation among *CpbHLH* subfamilies (Figure 4C).

Gene duplication events are crucial in the evolutionary process and contribute to the generation of new genes [61,62,63,64]. These events have greatly facilitated the diversification of gene families [65]. In the case of *CpbHLH* genes, we identified five gene pairs that were tandem duplicates (Figure 1), while 56 gene pairs were the result of segmental duplications (Figure 5A). This indicates a significant contribution of gene duplication events, particularly segmental duplications, as a driving force behind the expansion of the *CpbHLH* gene family during evolution. Similar patterns have been observed in the bHLH gene families of *P. mume* and *Fagopyrum tataricum* (L.) Gaertn. (Polygonaceae) [56,66]. The Ka/Ks ratios of these duplicated gene pairs of *CpbHLH* suggested that this gene family experienced purifying selection, a clear indication of highly conserved evolution. In the collinearity analysis, we found that *CpbHLH* genes showed a high level of collinearity with *P. trichocarpa* bHLH genes, followed by *V. vinifera*, *O. sativa*, and *A. thaliana*, indicating that these bHLH genes probably descended from a common ancestor (Figure 5B). Moreover, the presence of a few collinear genes across the genomes of all the tested plants suggests that these genes may hold significant importance in the evolutionary dynamics.

To comprehensively investigate the function of the bHLH family in Wintersweet, we performed an in-depth analysis of the expression patterns of *CpbHLH* genes across various tissues. A total of 94 *CpbHLH* genes were found to be expressed in at least one tissue, and their expression levels varied greatly across tissues (Table S8). Some *CpbHLH* genes exhibited high expression levels in specific tissues, indicating potential roles in those tissues’ development (Figure 6A). Considering the conserved properties of gene families, the putative functions of *CpbHLH* genes could be predicted based on their orthologous genes [67]. The combination of phylogenetic tree and expression analyses served as a foundation for further investigations. Notably, the expression levels of *CpbHLH87*, *CpbHLH82*, *CpbHLH88*, *CpbHLH6*, *CpbHLH17*, *CpbHLH73*, *CpbHLH103,* and *CpbHLH67* genes were higher in flowers compared to other tissues (Figure 6A), suggesting their potential importance in flower development. For instance, *CpbHLH73* clustered with *AtbHLH24* (SPATULA) in the VII(a+b) subfamily (Figure 3), and it has been reported that *AtbHLH24* regulates organ morphogenesis (carpel development) [19]. Moreover, the transcript abundance of *CpbHLH114*, *CpbHLH57,* and *CpbHLH10* was highest in leaves (Figure 6A). In the phylogenetic tree, *CpbHLH114*, *CpbHLH57,* and *CpbHLH10* clustered with *AtbHLH97*, *AtbHLH45,* and *AtbHLH98* in the Ia subfamily, which regulates the stomata development in Arabidopsis [68], suggesting a potential similar function in Wintersweet.

Next, we delved deeper into the expression profile of *CpbHLH* genes during the development of Wintersweet flowers, aiming to gain insights into their roles in Wintersweet flowers. In total, 97 *CpbHLH* genes were expressed in at least one stage of flower development, with 77 differentially expressed genes and 20 genes showing stable expression across three flower development stages in two Wintersweet genotypes (Tables S6 and S7), indicating the potential involvement of *CpbHLH* genes in Wintersweet flower development. Particularly, *CpbHLH86*, *CpbHLH15*, and *CpbHLH110* exhibited differential expression with high levels during the early stages of flower development, and the expression levels of these genes, especially *CpbHLH15* and *CpbHLH86*, were similar in both genotypes, suggesting conserved functions in Wintersweet (Figure 6B and Figure 7). In the phylogenetic tree, *CpbHLH110* clustered with *AtbHLH10*, *AtbHLH89*, *AtbHLH91*, and *AtbHLH138* in subfamily II, while *CpbHLH15* closely clustered with *AtbHLH21* in subfamily III (a+c) (Figure 3). These genes have been reported to be required for anther development in *Arabidopsis* [69,70]. Furthermore, *CpbHLH86* clustered with Cryptochrome interacting bHLHs (CIBs) in subfamily XII, which act as positive regulators in CRY2-mediated flowering time in *Arabidopsis* [71], suggesting a potential role for *CpbHLH*s in a similar process in Wintersweet.

*CpbHLH118*, *CpbHLH66,* and *CpbHLH96* clustered with *AtbHLH1*, *AtbHLH2*, *AtbHLH12,* and *AtbHLH42* in IIIf subfamily (Figure 3), which have been associated with anthocyanin and proanthocyanidin biosynthesis, trichome formation and root hair patterning [72,73,74]. In this study, *CpbHLH118*, *CpbHLH66,* and *CpbHLH96* showed differential expression during flower development, and their expression patterns also differed between the two genotypes. Specifically, *CpbHLH118* and *CpbHLH66* exhibited a descending expression trend during flower development in HLT040 genotype (dark yellow petals), while in the HLT015 genotype (red middle petals), their expression significantly upregulated at the open flower stage compared to HLT040 genotype (Figure 6B), This suggests that they may play a role in regulating floral color variation between the two genotypes. Previous studies have demonstrated that *CpbHLH1* (referred to as *CpbHLH118* in this study) and *CpTT8* (referred to as *CpbHLH96* in this study) act as negative and positive regulators of anthocyanin biosynthesis, respectively [18,40]. *CpbHLH66* shares sequence similarity with *CpbHLH118* but lacks the DNA binding region. However, it contains the conserved amino acid residues in helix regions required for dimerization, suggesting that *CpbHLH66* may form heterodimers with other transcription factors to enhance or repress their activity. Additionally, *CpbHLH87*, *CpbHLH77*, and *CpbHLH17* also showed differential expression during flower development and between the genotypes. *CpbHLH87* showed a higher expression level at bud stage 2 in the HLT015 compared to the HLT040 genotype. It shares the highest sequence similarity with BIM1 (a key regulator of the brassinosteroid signaling), and previous reports have shown that brassinosteroid, along with other phytohormone signaling, enhances anthocyanin accumulation in plants, potentially regulated using ternary MYB-bHLH-WD transcriptional complexes [75,76]. However, further functional validation is required to confirm the specific roles of these *CpbHLH* genes.

Compared to other TFs, bHLH TFs exhibit versatile regulatory functions, participating in multiple pathways and acting as co-regulators of gene expression alongside other proteins [77]. Previously, it has been revealed that heterologous expression of Wintersweet bHLH13 (referred to as *CpbHLH25* in this study) and MYC2 (referred to as *CpbHLH59* in this study) in model plants (*Arabidopsis* and tobacco) increased the *β*-caryophyllene and Linalool contents, respectively, a reduction in anthocyanin content was also observed in bHLH13 overexpressing plants [41]. In the present study, protein–protein interaction assays were conducted, confirming the physical interaction between CpbHLH25 and CpbHLH59 (Figure 8). Furthermore, the interaction of CpbHLH25 and CpbHLH59 with other proteins, specifically CpbHLH112 and CpMYB2, was also observed (Figure 8). Qian et al. (2021) reported that CpMYB2 interaction with CpTT8 is required for the activation of the anthocyanin biosynthetic structural gene *CpANS* [40]. Furthermore, Shang et al. (2020) previously suggested that *CpbHLH112* is a potential candidate for regulating scent biosynthesis in Wintersweet flowers [42]. Therefore, the observed physical interactions among CpbHLH25, CpbHLH59, CpbHLH112, and CpMYB2 indicate their coordinated involvement in the regulation of secondary metabolite biosynthesis in Wintersweet flowers.

## 4. Materials and Methods

### 4.1. Retrieval of CpbHLH Family Genes

The Wintersweet genome sequences and protein sequences, along with annotation information, were acquired from the Wintersweet genome database [42]. The HMMER 3.0 software program [78] was employed to search for potential *CpbHLH* candidates in the Wintersweet genome database using the hidden Markov Model (HMM) profile of the HLH (PF00010) domain that was obtained from the Pfam database [79]. All of the retrieved CpbHLH protein sequences were further examined using NCBI CDD [80] and SMART [81] databases to verify the presence of the bHLH domain. To ensure the identification of all *CpbHLH*s as well as possible, genes containing incomplete or atypical bHLH structural domains were also retained for subsequent analysis. The physicochemical characteristics of CpbHLH proteins, such as the numbers of amino acids (aa), isoelectric point (pI), and molecular weights (MW), were determined using the ExPASy (ProtParam) online tool (https://web.expasy.org/protparam/ (accessed on 3 January 2023)). The subcellular location of the CpbHLH protein was predicted using WoLF PSORT (https://www.genscript.com/wolf-psort.html (accessed on 3 January 2023)). Furthermore, the sequences of conserved domains in CpbHLH proteins were visualized and analyzed using the Weblogo3 and Jalview software (version 2.11.2.7).

### 4.2. Conserved Motif and Intron/Exon Organization of CpbHLHs

MEME SUITE (https://meme-suite.org/meme/tools/meme (accessed on 8 January 2023)), an online tool, was used to examine the motifs (max. number = 20) of the CpbHLH proteins [82]. Gene structure (intron/exon) information was retrieved from the generic feature format (GFF) file of the Wintersweet genome database [42]. The schematic representation of protein motifs and intron/exon organization was drawn using the gene structure view (advanced) program embedded in TBtools.

### 4.3. Phylogenetic Analysis and Chromosomal Distribution of CpbHLHs

The protein sequences of *A. thaliana* bHLH were retrieved from the TAIR database. Multiple sequence alignment of *CpbHLH*s, *AtbHLH*s, and a few functionally characterized bHLHs was performed using the ClustalW algorithm in MEGA7 software (version 7.0.21) with default parameters. The neighbor-joining (NJ) phylogenetic tree was constructed using pairwise deletion, *p*-distance method, and 1000 bootstrap replication in MEGA7. NJ phylogenetic tree of *CpbHLH*s was constructed using the same parameters as described above. The Newick extension of the MEGA NJ was used as a query to obtain the final figure of the NJ tree using the iTOL. The positions of *CpbHLH* genes on specific chromosomes were visualized using TBtools software (version v2.001) based on the chromosomal location information of genes obtained from the GFF file of the Wintersweet genome database.

### 4.4. Promoter Analysis of CpbHLHs

We obtained the 2 kb upstream sequence of the start codon of each *CpbHLH* gene from its full-length genomic sequence using TBtools and subjected it to the PlantCARE database for the prediction of putative cis-acting elements [83].

### 4.5. Gene Duplication Events and Syntenic Analysis

To detect the gene duplication events of bHLH genes within *C. praecox*, and to find the homology of the bHLH genes between *C. praecox* and the other selected plants (*A*. *thaliana*, *P*. *trichocarpa*, *V*. *vinifera,* and *O*. *sativa*), the MCscanx tool embedded in TBtools was used. The Ka/Ks calculator integrated in TBtools was employed to examine the Ka and Ks values of duplicated *CpbHLH* gene pairs, and the Ka/Ks ratio was calculated to evaluate the selection pressure. The genomic information of *A*. *thaliana*, *P*. *trichocarpa*, *V*. *vinifera,* and *O*. *sativa* was downloaded from Phytozome.

### 4.6. Plant Material

Two genotypes of Wintersweet (HLT015 and HLT040), grown under natural conditions in Heilongtan Park (Kunming, China), were selected. The morphological attributes of these genotypes were as follows: (1) HLT015, light yellow-colored flowers with red inner petals; (2) HLT040, dark yellow-colored flowers with yellow petals. For RNA sequencing, three biological replicates of green buds (Stage 1), buds turning yellow (Stage 2), and open flowers (Stage 3) were collected from both genotypes. For tissue-specific expression analysis, cotyledon, leaf, root, and flower tissues of Wintersweet were collected. All samples were immediately frozen in liquid nitrogen and stored at −80 °C until RNA extraction.

### 4.7. Expression Profile of CpbHLHs Based on Transcriptome Database

The FPKM (Fragments Per Kilobase per Million mapped reads) values of *CpbHLH* candidate genes were retrieved from the transcriptome databases generated from three developmental stages of flower (green bud, bud turning yellow, and open flower stage) of two Wintersweet genotypes as well as different organs (cotyledon, root, leaf and flower) of Wintersweet plant. We used TBtools to create heat maps that show the transcript profiles of *CpbHLH* genes based on the FPKM values.

### 4.8. Quantitative Real-Time PCR (RT-qPCR)

To validate the transcriptome results, we used RT-qPCR to measure the transcription levels of the differentially expressed genes (DEGs) with gene-specific primers. The extraction of total RNA from two Wintersweet genotypes flowers at three developmental stages (S1: green buds, S2: buds turning yellow, and S3: open flowers) was carried out using the EASYspin plant RNA extraction kit (aidlab, Beijing, China) as per manufacturer’s protocol. Subsequently, the integrity and purity of RNA were determined using a 1.5% denaturing agarose gel and a Nanodrop-2000 spectrophotometer, respectively. The reverse transcription of RNA into cDNA was carried out using the cDNA Synthesis SuperMix (TransGen Biotech, Beijing, China) according to the manufacturer’s instructions. RT-qPCR was performed in a 20 µL reaction volume comprising Blastaq^TM^ 2X qPCR MasterMix (10 µL), cDNA (1 µL, 100 ng/µL), forward primer (0.5 µL), reverse primer (0.5 µL) and ddH2O (8 µL) on the Roche LightCycler^R^ 480 II Real-Time PCR platform. The experiments were conducted in triplicates, and *β*-actin was used as an internal reference for normalization. Finally, the 2^−ΔΔCT^ method was utilized for the calculation of relative gene expression levels. The primers used in this study are listed in Table S5.

### 4.9. Yeast Two-Hybrid Assay (Y2H)

The open reading frames (ORFs) of CpbHLH25, CpbHLH59, CpMYB2, and CpbHLH112 were amplified using gene-specific primers and cloned separately into pGBKT7 and pGADT7 vectors to generate bait and prey constructs, respectively. We then co-transformed the respective pairs of recombinant vectors into the AH109 yeast strain and performed the Y2H assay as directed by the manufacturer (Clontech). The transformants (AH109 cells) carrying the pairs of recombinant vectors were cultured on synthetic dropout media SD/-Leu-Trp, and the pairs interactions were evaluated on the SD/-Leu-Trp-His-Ade + 3-amino-1,2,4-triazole (3-AT) (40 mM/L) + X-α-gal (25 mg/L) media.

## 5. Conclusions

In the current study, we performed a comprehensive genome-wide analysis of the bHLH gene family in Wintersweet. Our analysis revealed the presence of 131 *CpbHLH* genes distributed across the 11 chromosomes of Wintersweet, which were classified into 23 distinct subfamilies. The accuracy of this classification was further supported using motif and gene structure analyses. Notably, gene duplication, particularly segmental duplication, emerged as a key driver of bHLH gene diversity. RNA-seq analysis illuminated distinct expression patterns in various tissues and during flower development, highlighting intricate transcriptional regulation. Furthermore, CpbHLH25 and CpbHLH59 (regulators of floral scent and color) form heterodimers not only with each other but also with CpbHLH112 and CpMYB2, suggesting their potential coordination in regulating secondary metabolite biosynthesis. Overall, these findings offer valuable insights for future research investigating the biological functions of bHLH gene family members in Wintersweet flowers.

## Data Availability

Not applicable.

## Supplementary Materials

The following supporting information can be downloaded at: https://www.mdpi.com/article/10.3390/ijms241713462/s1.
