# Supplementary material for: Genome-Wide Identification and Expression Analysis of the bHLH Transcription Factor Family in Wintersweet (Chimonanthus praecox)
Source: Int J Mol Sci. 2023 Aug 30;24(17):13462. doi: 10.3390/ijms241713462 (PMC10487621; doi:10.3390/ijms241713462)
Supplement: Supplementary file 1 [file ijms-24-13462-s001.zip › Supplementary figures.pdf]

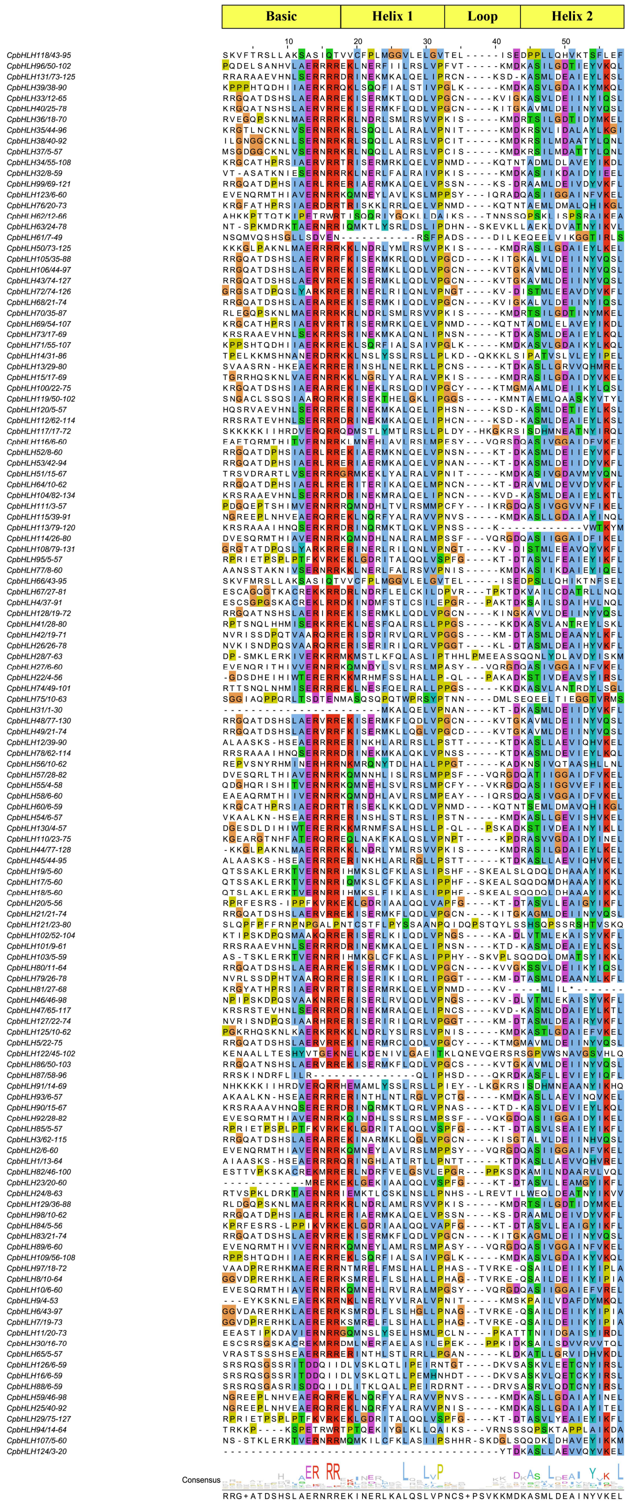

Figure S1. Partial display of sequence alignment of the CpbHLH proteins by Jalview. The capital letters of alphabet indicate the amino acids with more than 50% conservation.

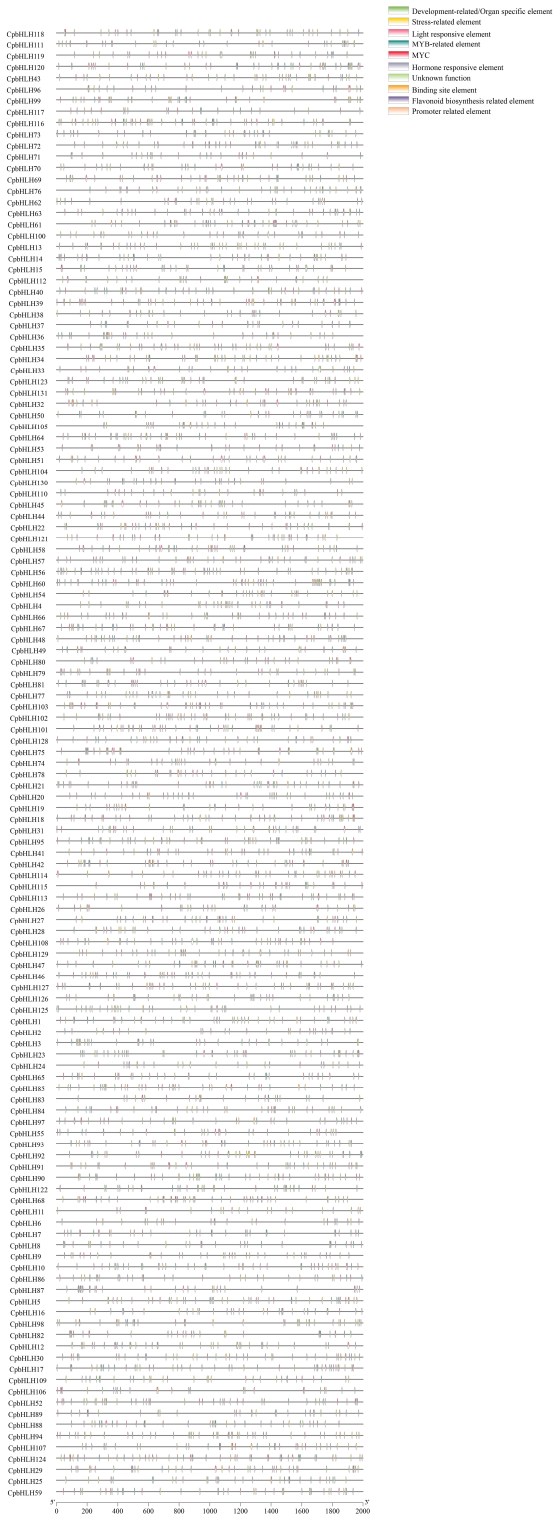

Figure S2. *Cis*-regulatory elements in the promoter regions of the *CpbHLH* genes. Different *cis*-regulatory elements are represented with different colored boxes, which are placed at the top on the right. The element size is estimated by the scale at the bottom
